# Chir99021 and Valproic acid reduce the proliferative advantage of *Apc* mutant cells

**DOI:** 10.1038/s41419-017-0199-9

**Published:** 2018-02-15

**Authors:** Alistair J. Langlands, Thomas D. Carroll, Yu Chen, Inke Näthke

**Affiliations:** 1 Cell & Developmental Biology, School of Life Sciences, University of Dundee, Dundee, DD1 5EH Scotland; 2 Present Address: National Phenotypic Screening Centre, School of Life Sciences, University of Dundee, Dundee, DD1 5EH Scotland

## Abstract

More than 90% of colorectal cancers carry mutations in *Apc* that drive tumourigenesis. A 'just-right' signalling model proposes that *Apc* mutations stimulate optimal, but not excessive Wnt signalling, resulting in a growth advantage of *Apc* mutant over wild-type cells. Reversal of this growth advantage constitutes a potential therapeutic approach. We utilised intestinal organoids to compare the growth of *Apc* mutant and wild-type cells. Organoids derived from *Apc*^*Min/+*^ mice recapitulate stages of intestinal polyposis in culture. They eventually form spherical cysts that reflect the competitive growth advantage of cells that have undergone loss of heterozygosity (LOH). We discovered that this emergence of cysts was inhibited by Chiron99021 and Valproic acid, which potentiates Wnt signalling. Chiron99021 and Valproic acid restrict the growth advantage of *Apc* mutant cells while stimulating that of wild-type cells, suggesting that excessive Wnt signalling reduces the relative fitness of *Apc* mutant cells. As a proof of concept, we demonstrated that Chiron99021-treated *Apc* mutant organoids were rendered susceptible to TSA-induced apoptosis, while wild-type cells were protected.

## Introduction

The Wnt signalling pathway is an important regulator of intestinal crypt homoeostasis. It is required for maintenance of intestinal stem cells^1,2^, and regulates proliferation, migration and differentiation^3^ in intestinal crypts. Activating the Wnt pathway is strongly associated with colorectal cancer. Indeed, >90% of all colorectal cancers carry mutations in *Adenomatous polyposis coli* (*Apc*)^4^, which increases Wnt signalling.

Mice and patients heterozygous for mutations in *Apc* invariably develop intestinal polyps. Where in the intestinal-tract-specific *Apc* mutations produce lesions most commonly is likely related to the endogenous Wnt signal that exists locally plus the increased signal generated by the mutation^5^. Endogenous Wnt signalling changes along the proximal distal axis of the intestinal tract^6,7^. Together with predicted differences in Wnt signalling changes produced by different *Apc* mutations, these ideas have led to the 'just-right' hypothesis^8,9^. It postulates that specific *Apc* mutations are selected to provide optimal Wnt signalling and to provide the greatest proliferative advantage over surrounding wild-type tissue.

Inhibitors of Wnt signalling are being considered as potential anti-cancer treatments. They aim to inhibit the advantage conferred by optimal Wnt signalling on *Apc* mutant cells^10^. For such therapies to be successful, it is crucial to understand how modifying Wnt signalling affects the relative growth advantage of *Apc* mutant cells over wild-type cells. Other signalling pathways, particularly Notch and BMP, also contribute to responses of cells to Wnt, complicating the situation and making it difficult to study how single factors affect relative growth, particularly in whole animals (reviewed in refs.^11,12^).

Intestinal organoids provide a useful model system to investigate the relative growth advantage of different cells in the context of the intestinal epithelium^13^. They constitute a powerful tool for measuring the effect of potential cancer therapies, without the ethical and cost implications of animal experimentation^13^. The *Apc*^*Min/+*^ mouse has been used extensively to study early stages of cancer^14,15^. Organoids derived from *Apc*^*Min/+*^ mice recapitulate polyp development in vivo; they initially maintain normal crypt–villus architecture, but eventually grow as spherical cysts reflecting the selective growth advantage of cells that have undergone LOH^16^. Cells in these spherical *Apc*^*Min/Min*^ organoids have prominent nuclear β-catenin, consistent with elevated Wnt signalling.

Here, we demonstrate that treatment with the small molecule inhibitors, Chiron99021 (C) and Valproic acid (V), reduces the growth advantage of *Apc* mutant cells at concentrations that activate Wnt and Notch signalling^17^. We found that CV treatment prevented the transformation of *Apc*^*Min/+*^ to *Apc*^*Min/Min*^ organoids. The latter grew relatively more slowly in CV, but there was no increase in apoptosis. Sensitising *Apc*^*Min/Min*^ organoids by potentiating Wnt signalling rendered them more susceptible to TSA-induced death, while it protected wild-type organoids. These results provide a proof-of-concept for the idea that excessive Wnt stimulation can reduce the fitness of *Apc* mutant cells and render them more susceptible to death.

## Results

### Characterisation of *Apc*^*Min/+*^ organoids

**Fig. 1:**
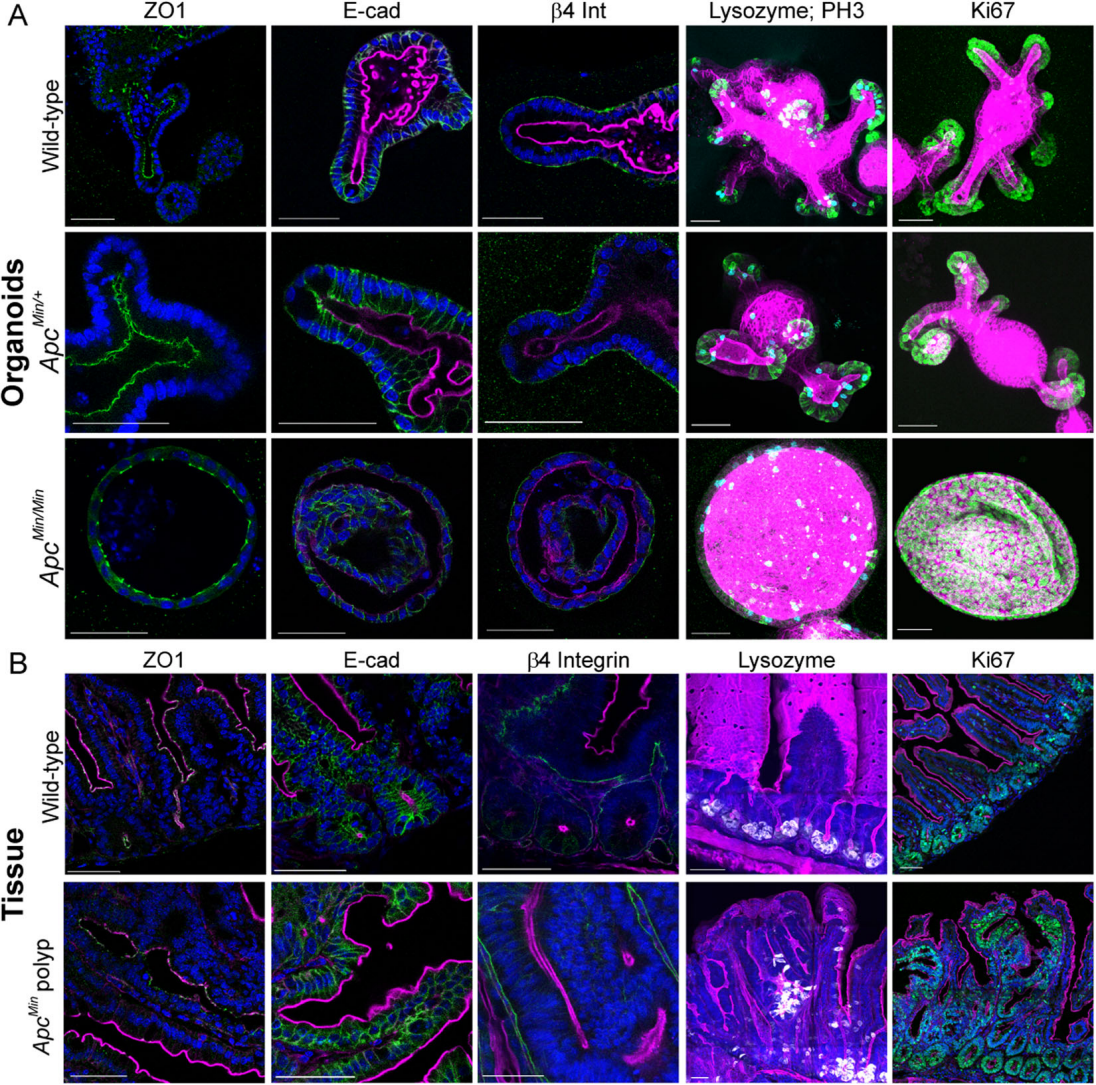
*Apc*^*Min/Min*^ organoids maintain epithelial polarity but lose tissue organisation. **a** Apical-basal polarity is maintained in *Apc*^*Min/+*^ organoids as revealed in single optical sections of wild-type (upper panel), *Apc*^*Min/+*^ (middle panel) and *Apc*^*Min/Min*^ (lower panel) organoids stained with Hoechst (blue) and Phalloidin (magenta) to visualise nuclei and actin; ZO1 (green, left panel), E-cadherin (green, middle panel) and β4 integrin (green, right panel) mark apical, lateral and basal membranes, respectively. Crypt–villus organisation is lost in *Apc*^*Min/Min*^ organoids, as revealed in maximum intensity projections (far right panels) of organoids stained to reveal actin (Phalloidin, magenta); Lysozyme (Paneth cells, green), PH3 (mitotic cells, cyan), left panel; and Ki67 (proliferative cells, green), right panel. *Apc*^*Min/+*^ organoids were imaged in the first three passages after isolation, *Apc*^*Min/Min*^ were imaged after at least five passages. Scale bars = 50 µm. **b** Cryo-sectioned intestinal tissue from wild-type mice (upper panel) and from polyps in *Apc*^*Min/+*^ mice (lower panel) stained with Hoechst (blue) and Phalloidin (magenta). Localisation of polarity markers (green: ZO1, left panel; E-cad, middle panel; and β4 integrin, right panel) reveals that epithelial polarity is maintained in polyps. Vibratome-sectioned intestinal tissue stained to detect Lysozyme (white) reveals Paneth cells at the crypt base in wild-type tissue, but distributed throughout polyps. Cryo-sectioned intestinal tissue stained against Ki67 (green) reveals that proliferative epithelial cells are restricted to crypts in wild-type tissue, while almost all cells in a polyp are proliferative. Scale bars = 100 µm

We first established that organoids were appropriate surrogates for tissue in situ by examining whether they recapitulate polyp development. Organoids isolated from *Apc*^*Min/+*^ mice were initially indistinguishable from wild-type organoids, but after a few passages they grew as spherical cysts that only contained cells that had undergone LOH (Fig. 1; Figure S1A-C) as reported previously^16^. The wild-type and mutant alleles of *Apc* were consistently detected in *Apc*^*Min/+*^, but only the mutant form was detected in *Apc*^*Min/Min*^ cysts (Figure S1A). Similar to *Apc*^*fl/fl*^ cysts^18^, epithelial polarity was maintained in *Apc*^*Min/Min*^ cysts, as revealed by the presence of apical ZO1, lateral E-cad and basal β4 integrin (Fig. 1a). However, the distribution of the different cell types normally found in the intestinal epithelium was altered. In both wild-type and *Apc*^*Min/+*^ organoids, mitotic (phospho-histone3 positive, PH3+), and proliferative (Ki67+) cells localised exclusively to crypts and Paneth cells were restricted to crypt bases (Fig. 1a). In *Apc*^*Min/Min*^ cysts, most cells were proliferative (Ki67+), and mitotic and Paneth cells were distributed throughout the cyst (Fig. 1a). The maintenance of epithelial polarity and loss of tissue organisation was similar to that in polyps in situ in *Apc*^*Min/+*^ mice (Fig. 1b), which represent tissue formed by cells that have undergone LOH in *Apc*. These data confirm that cells which have undergone LOH in organoids and tissue form similar structures.

Mutations in *Apc* lead to elevated Wnt signalling in polyps in *Apc*^*Min/+*^ mice. The accumulation of nuclear β-catenin in *Apc*^*Min/Min*^ suggests that the same is true in organoids^16^. Increased Wnt signalling in *Apc*^*Min/Min*^ cysts was confirmed by an increase in the amount of β-catenin protein (Figure S1B, C) and elevated expression of mRNA-encoding *Axin2*, *CycD* and *cMyc* (Figure S1D). None of the examined genes were altered in *Apc*^*Min/+*^ organoids relative to wildtype while they exhibited normal organoid morphology (Figure S1D). In tissue homozygous mutant for *Apc*, the number of differentiated intestinal cells is reduced^19^. Consistently, expression of differentiated cell markers was reduced in *Apc*^*Min/Min*^, but not *Apc*^*Min/+*^ organoids (Figure S1D, E), confirming that mutation of both *Apc* alleles is required to induce tumourigenic changes.

Other reported changes in *Apc* mutant tissue include elevated expression of EMT markers^20,21^. Consistently, we found that in *Apc*^*Min/Min*^ cysts, mRNA for both *N-cad* and *Vim* was also increased (Figure S1F). However, *E-cad* remained more highly expressed than *N-cad* and the ratio of *E-cad:N-cad* in *Apc*^*Min/Min*^ was similar to that in wild-type organoids. In comparison, in mouse fibroblasts, representative of mesenchymal cells, the *E-cad:N-cad* ratio was much lower (Figure S1G). Consistent with their epithelial characteristics, *Apc*^*Min/Min*^ cysts maintained normal levels of E-cad protein (Figure S1H, I).

Together, these results confirmed that *Apc*^*Min/Min*^ cysts are phenotypically similar to polyps that appear in *Apc*^*Min/+*^ intestinal tissue and reflect structures formed by cells that had undergone LOH, validating them as a suitable model for early tumourigenesis.

### Chiron99021 and Valproic acid prevent tumourigenic transformation of *Apc*^*Min/+*^ organoids

**Fig. 2:**
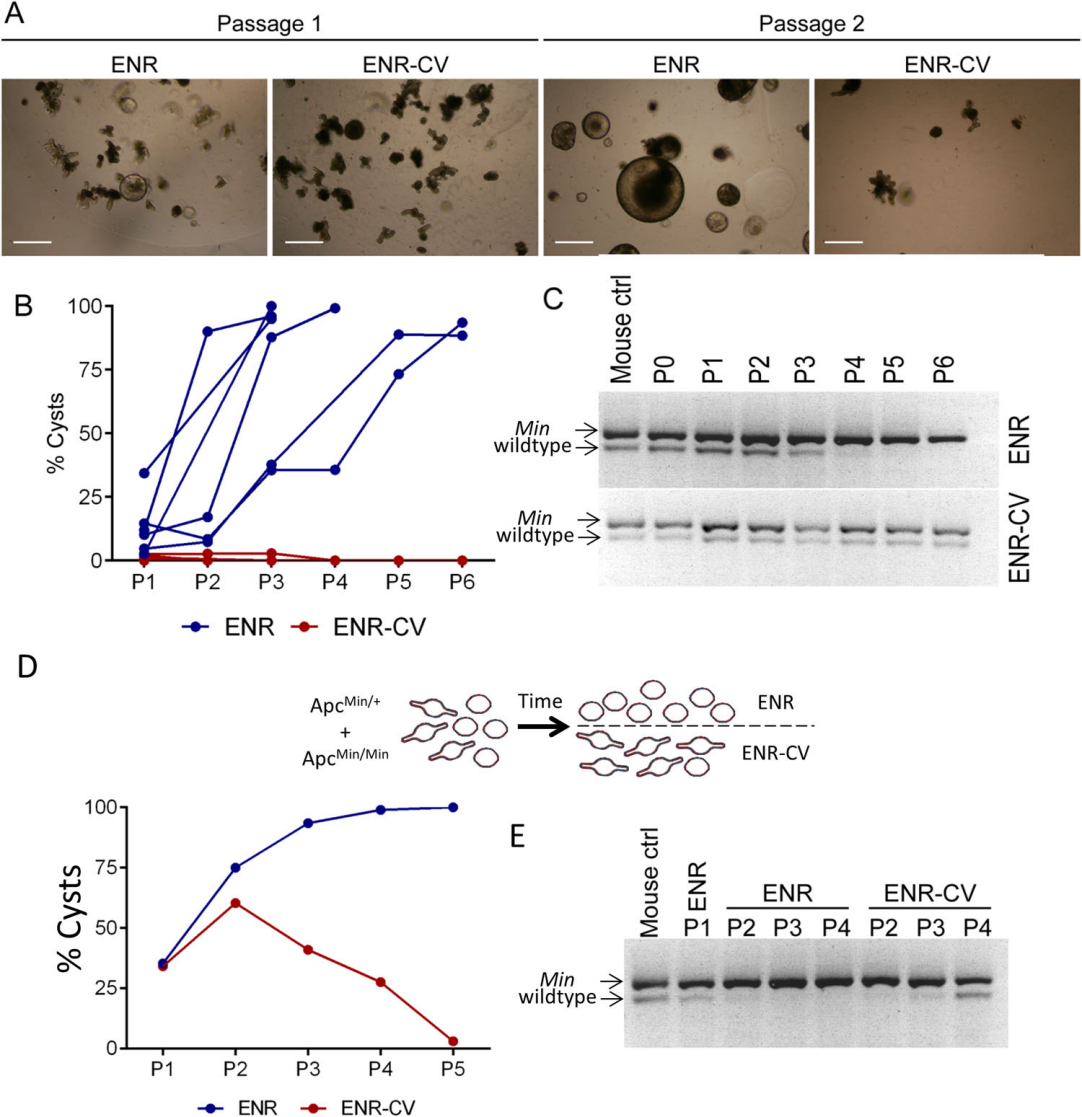
Chiron99021 and Valproic acid inhibit formation of *Apc*^*Min/Min*^ cysts. **a** Images of organoids derived from *Apc*^*Min/+*^ after one (left) and two (right) passages in ENR or ENR-CV. **b** Proportion (%) of total organoids exhibiting cyst morphology at the end of each passage in ENR and ENR-CV. **c** Genotyping of organoids at the end of each passage shows that *Apc*^*Min/+*^ organoids grown in ENR-CV maintain the wild-type *Apc* allele while only the mutant allele is detectable in ENR. **d** Equal numbers of wild-type and *Apc*^*Min/Min*^ organoids were mixed, and the percentage of organoids growing as cysts was determined after successive passages. **e** Genotyping of the mixed populations of wild-type and *Apc*^*Min/Min*^ organoids shown in (**d**) confirms the disappearance of the wild-type allele in ENR and its re-emergence in ENR-CV corresponding to the relative abundance of cysts in each case

Chiron99021 and Valproic acid are commonly included in early passages for organoid culture because they improve the efficiency of organoid formation. They increase Wnt and Notch signalling resulting in the presence of more Lgr5+ stem cells^17^. Surprisingly, these reagents prevented the transformation of *Apc*^*Min/+*^ organoids. *Apc*^*Min/+*^ organoids robustly formed spherical cysts after repeated passage in normal (ENR) media. However, when grown in normal media (ENR) supplemented with Chiron 99021 and Valproic acid (ENR-CV), they maintained normal morphology (Fig. 2a, b). Genotyping revealed that the wild-type allele persisted in ENR-CV but was lost in ENR (Fig. 2c). We wanted to determine whether a reduction in the rate of LOH or a shift in the relative growth advantage of *Apc*^*Min/+*^ and *Apc*^*Min/Min*^ cells was responsible for these observations. The number and size of polyps in *Apc*^*Min/+*^ mice increase with age reflecting the increasing number of LOH events in the tissue^22^. If cyst formation reflects the relative outgrowth of *Apc*^*Min/Min*^ present at the time of tissue isolation, cysts should appear more rapidly in organoid cultures established from older mice. Indeed, organoids isolated from ~60-day-old *Apc*^*Min/+*^ mice transformed into cysts less frequently than those isolated from ~90-day-old *Apc*^*Min/+*^ mice (Figure S2). These observations are consistent with the idea that *Apc*^*Min/Min*^ cysts in culture emerged from *Apc*^*Min/Min*^ cells that had undergone LOH in the animal before tissue isolation. This is also consistent with the observation that *Apc*^*Min/+*^ organoids are genetically stable in culture^23^ and suggests that Chiron 99021 and Valproic acid reduces the growth advantage of *Apc*^*Min/Min*^ cells.

To test this idea further, we mixed *Apc*^*Min/+*^ and *Apc*^*Min/Min*^ organoids, and compared the growth in ENR and ENR-CV (Fig. 2d). After one passage in ENR, the wild-type allele was barely detectable suggesting that fewer heterozygous cells were present. However, the wild-type allele became more abundant again when the same organoids were transferred to and then passaged in ENR-CV, but not when they continued to grow in ENR (Fig. 2e). These data confirmed that ENR-CV altered the relative growth advantage of wild-type and *Apc*^*Min/Min*^ organoids.

**Fig. 3:**
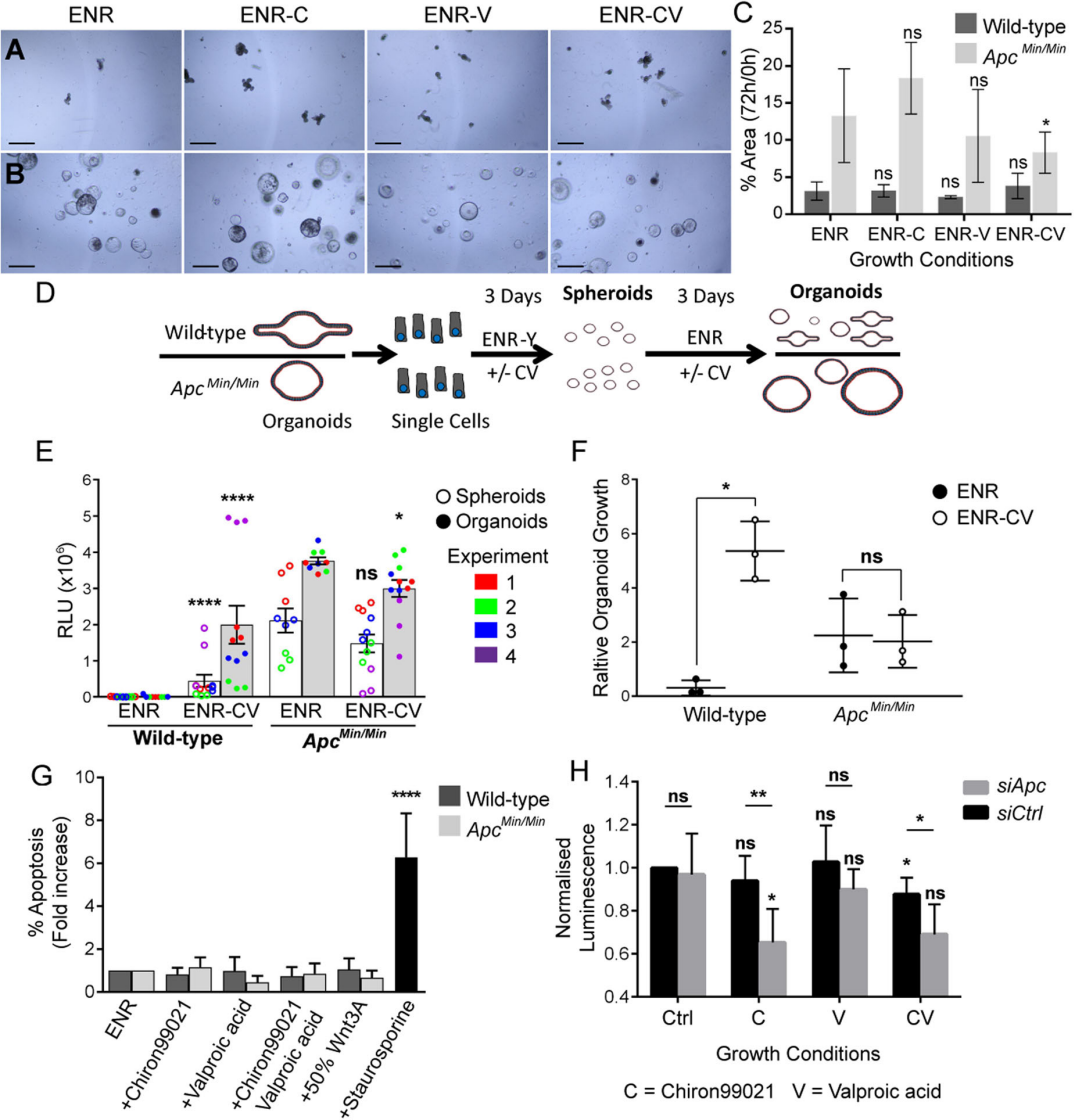
Growth of *Apc*^*Min/Min*^ organoids is slowed in ENR-CV. **a**,** b** Images of wild-type (**a**) and *Apc*^*Min/Min*^ (**b**) organoids after 72 h treated as indicated with either Chir99021 (‘ENR-C’) and/or Valproic acid (‘ENR-CV’ or ‘ENR-V’). **c** Quantifying organoid growth over 72 h revealed decreased growth of *Apc*^*Min/Min*^ organoids in ENR-CV only (*p* = 0.02), while growth of wild-type organoids was unaffected (average of three experiments ± SD, statistical significance for each condition compared to ENR by *t* test). **d** Schematic representation of experimental design to measure organoid recovery and growth after passaging to single cells using viability assays. Single cells from wild-type or *Apc*^*Min/Min*^ organoids were grown in ENR ± CV containing Y27632 for 3 days (at this point they only form spheroids representing early growth from single cells), and for a further 3 days in ENR ± CV without Y27632 to form organoids or cysts. Viability was determined by measuring relative luminescence intensity using CellTiterGLO, and measured for parallel cultures after 3 days and 6 days. Wells containing wildtype and *Apc*^*Min/Min*^ were set up in parallel. Three technical replicates were performed in each case and the results shown are from three (ENR) or four (ENR-CV) independent experiments. **e** Relative luminescence intensity of wild-type and *Apc*^*Min/Min*^ spheroids (white bars) and organoids/cysts (grey bars) were measured as described in (**d**). Initial growth in ENR-CV significantly increased the viability of wild-type spheroids (*p* < 0.0001) and organoids (*p* < 0.0001), but did not significantly change the viability of *Apc*^*Min/Min*^ spheroids (*p* = 0.6172) and reduced the viability of *Apc*^*Min/Min*^ cysts (*p* = 0.0207). These data show that wild-type spheroids form and grow to organoids much more readily in ENR-CV than in ENR, while *Apc*^*Min/Min*^ spheroid formation and growth to cysts is slightly reduced under these conditions. Bar graph shows average values ± SEM. Kolmogorov–Smirnov tests to compare distribution of values were performed to assess statistical significance. **f** Relative growth of wildtype and *Apc*^*Min/Min*^ from spheroids to organoids. Relative organoid growth was calculated by dividing the average luminescence intensity of organoids (grey bars in **e**) by the average luminescence intensity of the preceding spheroid stage for each of three independent experiments (values represented by white bars in **e**). ENR-CV increased the growth of wild-type organoids (*p* = 0.0114), but did not significantly change the growth of *Apc*^*Min/Min*^ cysts (*p* = 0.8301). Graph shows values ± SD, Welch’s *t* tests were performed to calculate statistical significance. **g** Wild-type and *Apc*^*Min/Min*^ organoids were treated with ENR-C, ENR-V, ENR-CV or ENR + 50% Wnt3A for 48 h and apoptosis determined by measuring the number of cells with active caspase-3 using flow cytometry. Data from four independent experiments normalised to the percentage of apoptosis in ENR are shown. There was no significant increase in apoptosis (*p* > 0.05 compared to ENR for all conditions). Staurosporine was used as a positive control and increased apoptosis in wild-type organoids 6.2-fold (*p* < 0.0001). **h** Viability of HAB92 cells transfected with *siApc* or untargeted control (*siCtrl*) shows *siApc*-transfected cells were significantly less viable when treated with Chiron99021 compared to control media (DSMO, *p* = 0.02), and compared to *siCtrl-*transfected cells treated with Chiron99021 (*p* = 0.009). Chiron and Valproic acid reduced the viability of *siApc*-transfected cells compared to *siCtrl*-transfected cells (*p* = 0.03). The average of five independent experiments are shown ± SD. Statistics compared each condition to control for *siApc* and *siCtrl* (directly above bars), and *siApc* to *siCtrl* for each condition (above lines) by *t* test

The altered growth advantage in ENR-CV may be caused by increased growth of wild-type organoids, reduced growth of *Apc*^*Min/Min*^ cysts or both. To determine how ENR-CV alters growth advantage, we examined growth of wild-type and *Apc*^*Min/Min*^ organoids in ENR-CV, ENR-C, ENR-V and ENR. *Apc*^*Min/Min*^ cysts grew significantly more slowly in ENR-CV, while growth of wild-type organoids did not significantly change (Fig. 3a–c). The reduced growth of *Apc*^*Min/Min*^ cysts may be caused by reduced proliferation or an increase in apoptosis. Viability assays revealed that ENR-CV increased the formation and growth of wild-type organoids but slightly decreased the growth of *Apc*^*Min/Min*^ cysts (Fig. 3d–f). There was no increase in Caspase-3 activity in wild-type or *Apc*^*Min/Min*^ organoids grown in ENR-CV, suggesting that apoptosis was not affected (Fig. 3g). These data indicate that changes in proliferation are most likely responsible for the differential growth advantages of organoids in ENR-CV. To determine whether these changes in growth are recapitulated consistently in human cells, we compared the effect of Chiron99021 and Valproic acid on HAB92 colorectal cancer cells (a derivative of HCT116 cells, which are homozygous for wild-type β-catenin)^24^ transfected with *siRNA*-targeting *Apc* or a control (scrambled) *siRNA*. Consistent with our hypothesis, cells lacking APC were less viable than cells transfected with control *siRNA* when grown in Chiron99021 or Chiron99021 plus Valproic acid (Fig. 3h).

### The growth advantage of *Apc*^*Min/Min*^ cells reflects 'just-right' Wnt signalling

Gene expression analysis of organoids has shown that ENR-CV stimulates Wnt signalling^17^. Together with the 'just-right' Wnt signalling hypothesis^7^, this data prompted us to test whether reduced growth of *Apc*^*Min/Min*^ organoids in ENR-CV is related to activation of Wnt signalling beyond an optimal level. TOP/FOP assays performed in HEK293 cells confirmed that treatment with Chiron99021 alone, or Chiron99021 plus Valproic acid potently increased Wnt signalling, more so than treatment with Wnt3A-conditioned media (Figure S3A). The amount of β-catenin protein was increased when HAB92 cells were treated with Chiron99021 and/or Valproic acid (Figure S3B, D), including in cells depleted of APC (Figure S3D). Exogenous Wnt3A increased β-catenin protein levels in control *siRNA*-transfected HAB92 cells, although it did not increase β-catenin levels in cells depleted of APC (Figure S3C, E).

**Fig. 4:**
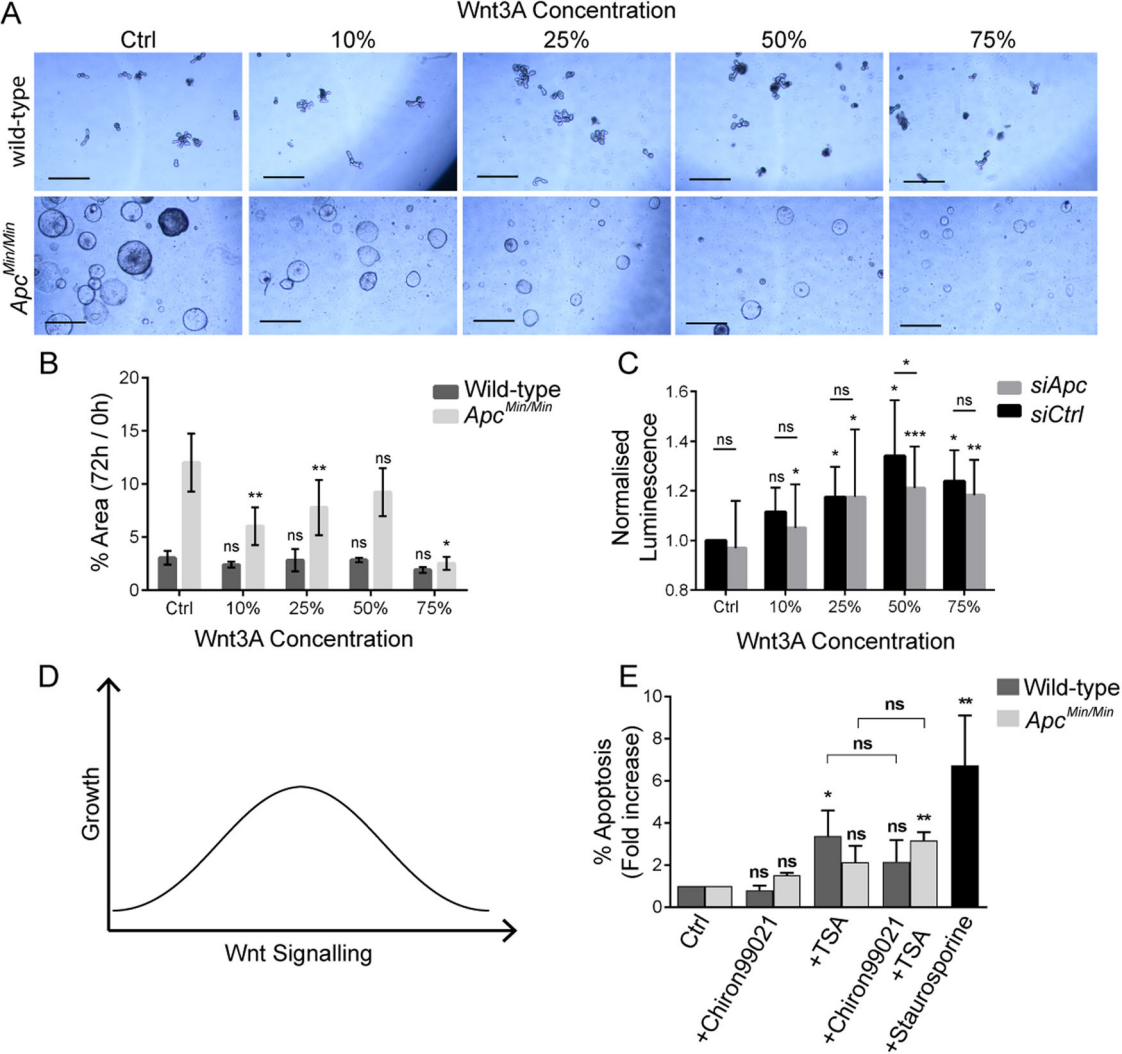
Increased Wnt signalling slows the growth of *Apc*^*Min/Min*^ cysts and sensitises them to TSA-induced apoptosis. **a** Wildtype (top panels) and *Apc*^*Min/Min*^ (bottom panels) show organoid cultures after 72 h growth in different concentrations of Wnt3A-conditioned media. Scale bars = 500 µm. **b** Measuring organoid growth over 72 h reveals that Wnt3A significantly slowed growth of *Apc*^*Min/Min*^ cysts (*p* = 0.008, 0.006, 0.12 and 0.02 for 10%, 25%, 50% and 75% Wnt3A, respectively), while not affecting the growth of wild-type organoids (*p* > 0.05 in all conditions). The average of three independent experiments show ± SD. Statistics compare growth in each concentration of Wnt3A-conditioned media to control conditions (ENR) by *t* test. **c** Viability of HAB92 cells was significantly increased by Wnt3A. The increase in viability was largest for *siCtrl*-transfected cell. At 50% Wnt3A, viability increased significantly more in *siCtrl* compared to *siApc* (*p* = 0.048). Average of five independent experiments show ± SD. Statistics compare each condition to control for siApc and siCtrl (directly above bars), and *siApc* to *siCtrl* for each condition (above lines) by *t* test. **d** Schematic model for how Wnt signalling affects growth. Wnt signalling increases growth until an optimal point is reached. Increasing Wnt beyond this optimal point reduces cell growth (e.g. *Apc*^*Min/Min*^ cysts grown in ENR-CV). **e** Relative increase in the proportion of apoptotic cells in wild-type and *Apc*^*Min/Min*^ organoids reveals that 24 h treatment with TSA increased apoptosis significantly only in wild-type organoids (*p* < 0.05). However, co-treatment with Chiron99021 protected wild-type organoids against this effect of TSA, but *Apc*^*Min/Min*^ cysts died more readily in response to TSA treatment when exposed to Chiron99021 (*p* < 0.05). Staurosporine increased apoptosis in wild-type organoids 6.74-fold (*p* < 0.01). Bar graph shows average values of three independent experiments ± SD normalised to percentage apoptosis in ENR

We then examined whether directly stimulating Wnt could alter the growth of organoids. Exposure to increasing amounts of Wnt3A-conditioned media slowed the growth of *Apc*^*Min/Min*^ cysts, but did not significantly change the growth of wild-type organoids (Fig. 4a, b). Similarly, treatment of HAB92 cells with Wnt3A-conditioned media increased the viability of cells transfected with control *siRNA* more than of those transfected with *siApc* (Fig. 4c). Differences in relative growth between the organoid and cell culture models may be explained by the incomplete knock-down of APC in the cell culture model (Figure S3D). Neverthless, these data support the idea that Wnt signalling above an optimal level could at least be partially responsible for the observed changes in organoid growth advantage. Treatment of organoids with Chiron99021 and Valproic acid may put Wnt signalling above the optimal level for *Apc*^*Min/Min*^, while putting Wnt signalling in wild-type or *Apc*^*Min/+*^ organoids closer to the optimal level (see schematic in Fig. 4d).

There did not appear to be a direct correlation between β-catenin protein level and cell viability measured in HAB92 cells (Fig. 4c; Figure S3E), suggesting that there is not a simple relationship between Wnt signal received, β-catenin protein level and cell growth. To understand whether this relationship could be explained by different transcriptional outputs, we compared the transcription of a panel of Wnt target genes in wild-type, *Apc*^*Min/+*^ and *Apc*^*Min/Min*^ organoids. We found that the expression of all the genes we analysed was indistinguishable between wild-type and *Apc*^*Min/+*^ organoids, but many were significantly upregulated or downregulated in *Apc*^*Min/Min*^ cysts (Figure S4). We then compared these genes in wild-type and *Apc*^*Min/+*^ organoids treated with Chiron99021 and/or Valproic acid. This analysis revealed a range of responses for different Wnt targets (Figure S4), suggesting a highly complex relationship between transcriptional responses, Wnt signalling and organoid growth that cannot be explained simply by the activity and function of Wnt target genes. In addition, many Wnt target genes respond to Valproic acid on its own (Figure S4). Thus identifying the specific Wnt targets responsible for the differential growth advantages of wild-type and *Apc* mutant cells under different growth conditions, will require a much more extensive and detailed analysis of the temporal dynamics and quantitative changes in transcriptional profiles.

### Potentiating Wnt signalling with Chiron99021 in *Apc* mutant organoids renders them more sensitive to TSA-induced death

Reagents that inhibit Wnt signalling are currently undergoing clinical trials as cancer therapies. However, their therapeutic usefulness has been questioned because of the potentially detrimental effects on wild-type cells^2,25^. Wild-type organoids but not *Apc*^*Min/Min*^ cysts died in the absence of the Wnt agonist R-Spondin (Figure S5A) and failed to grow in the presence of the Wnt inhibitor XAV-939, confirming the requirement of wild-type cells for Wnt (Figure S5B). The diminished growth advantage of *Apc*^*Min/Min*^ cysts that results from increasing Wnt signalling might facilitate the specific elimination of *Apc*^*Min/Min*^ cells. Indeed, we found that *Apc*^*Min/Min*^ cysts were more sensitive to the histone deacetylase inhibitor TSA than wild-type organoids in the presence of Chiron99021 (Fig. 4e), consistent with previous reports that TSA and SAHA (another histone deacetylase inhibitor) induce apoptosis in Wnt-activated colorectal cancer cell lines^26,27^. We compared the number of apoptotic cells in *Apc*^*Min/Min*^ and wild-type organoids treated with TSA and Chiron 99021. Strikingly, 24 h treatment of *Apc*^*Min/Min*^ cysts with TSA and Chiron99021 significantly increased the number of apoptotic cells, whereas wild-type organoids were not significantly affected (Fig. 4e). These data suggest that stimulating Wnt in *Apc* mutant cells can increase their susceptibility to TSA-induced death.

## Discussion

Before exploiting cancer associated changes in Wnt signalling, it is important to know how altering Wnt signalling affects wild-type and tumour cells. We show that treatment with the combination of Chiron99021 and Valproic acid reduces the proliferative advantage of *Apc* mutant cells, and propose that this may be facilitated through 'just-right' Wnt signalling. The novel concept that 'just-right' Wnt signalling can be manipulated to selectively eliminate *Apc* mutant cells is important to consider when developing therapeutic interventions that aim to eliminate *Apc* mutant cells in cancer.

In this context, it is noteworthy that an epidemiological study by the Danish Cancer Registry found no association between long-term use of the anti-depressant lithium, a GSK3 inhibitor and Wnt agonist, and increased risk of colorectal adenocarcinoma^28^. However, the region of the colon where cancer occurred in these patients was different than in patients not treated with lithium. This is consistent with the idea that the impact of increased Wnt signals varies depending on the levels of endogenous Wnt signalling present in different regions of the intestinal tract. Similarly, Lithium did not significantly increase polyp load in *Apc*^*Min/+*^ mice^29^ and the GSK3 inhibitor AZD7969 caused effects in dogs and rats that were completely reversible^30^. Together these observations suggest that increasing Wnt signalling may be a safe means to increase the susceptibility of tumour cells to targeted apoptosis.

Clinical exploitation of the 'just-right' Wnt signalling hypothesis will require examination of whether organoids derived from *Apc*^*Min/+*^ mice and human cancers respond similarly. Human colorectal cancers may contain any of a diverse range of Wnt-stimulating mutations^31^, and accumulate a multitude of additional mutations during development of the disease^32^, in contrast to the relatively simple LOH-induced formation of *Apc*^*Min/Min*^ cysts used to establish the principle in this study. Organoids derived from colorectal cancer patients have been shown to recapitulate several properties of the original tumour^33^, and could therefore allow testing of our hypothesis in a more clinically relevant model.

## Material and methods

### Ethics statement

All experiments involving animals were performed in accordance with UK Home office approved guidelines and were approved by the Home office Licensing committee (Project licenses PPL60/4172 and PPL70/8813).

### Organoid culture

Organoids were generated from mouse small intestinal crypts as described previously^13^. See Supplementary Information for a full description. Additional reagents (Chiron99012 [3 µM], Valproic acid [1 mM] and Y27632 [10 µM]; TSA, Wnt3A-conditioned media and XAV-939 [at indicated concentrations]) were added along with standard media. To genotype organoids, DNA was isolated by re-suspending one well of organoids in cold PBS, collecting organoids in a pellet by centrifugation (16,000 × *g*, 30 s) and extracting them in microLYSIS-Plus (Microzone) according to manufacturer’s instructions. Genotypes of organoids was determined using standard *Apc*^*Min/+*^ genotyping protocol (JAX lab). Organoid growth was determined by measuring organoid area as previously described^34^. Briefly, the average percentage area occupied by organoids was calculated from several non-overlapping images of organoids grown for 72 h, and divided by the average percentage area of freshly plated organoids (0 h) from the same well.

### Western blotting

Organoids or HAB92 cells were lysed in MEBC buffer (50 mM Tris-HCl, pH 7.5, 100 mM NaCl, 5 mM EGTA, 5 mM EDTA, 0.5% NP-40 and 40 mM β-glycerol phosphate), lysates separated on 4–12% SDS-PAGE (Invitrogen), and transferred to Protran nitrocellulose membrane (GE Healthcare). Immunoblotting was performed with primary antibodies listed in Supplementary Information and IRDye800/700-conjugated secondary antibodies (Rockland, 1∶5000) were detected with LiCor Odyssey Imager (Bioscience).

### qPCR

RNA was isolated using a Nucelospin kit (Machery Nagel) according to manufacturer’s instructions. A volume of 1 µg RNA was reverse transcribed using qscript cDNA Synthesis Kit (Quanta Biosciences), and 5 ng cDNA was used as template for amplification. An aliquot of 0.1 µM forward and reverse primers (Supplementary Information) were mixed with PerfeCTa SYBR Green Fastmix (Quanta Biosciences), and SYBR Green signal detected using CFX connect real-time qPCR system (Bio-Rad). Wnt target gene analysis (Figure S4) was carried out using WNT signalling targets (SAB Target List) M96 plates (Bio-Rad) according to manufacturer’s instructions.

### Cell culture

HEK293 and HAB92 cells were cultured in DMEM (Gibco) supplemented with 10% FBS (GE Healthcare) and 1% penicillin-streptomycin (Life Technologies). For TOP/FOP assays, HEK293 cells were plated at 5000 cells/well, grown for 1 day and transfected with TOP-Renilla or FOP-Renilla. After 1 day, fresh media containing Chiron99021, Valproic acid or Wnt3A conditioned media was added. Cells were incubated for 24 h, lysed and luciferase activity measured using Luciferase Assay System kit (Promega) according to manufacturer’s instructions. Control cells were transfected with empty pBluescript vector (negative control) or pCS2-β-catenin (positive control). Viability assays were performed using HAB92 cells transfected with 10 nM of *Apc*-targeting or control siRNAs (GE Healthcare) using INTERFERin (Polyplus) according to manufacturer’s instructions. Cells were grown for 48 h after transfection and passaged into 24-well plates at 10,000 cells per well. Cells were grown for 24 h in different media conditions as described, and viability measured using Cell TiterGLO (Promega) according to manufacturer’s instructions. Luminescence was measured using LumatLB luminometer (Berthold Technologies).

### Immunofluorescence

Immunofluorescence was performed as previously described for organoids^18^, cryo-sectioned intestinal tissue^35^ and vibratome-sectioned intestinal tissue^35^ using antibodies listed in Supplementary Information. Tissue and organoids were imaged with a Zeiss LSM 710 microscope (Carl Zeiss AG, Oberkochen, Germany) using ×40 Zeiss objective lens and immersion oil with a refractive index of 1.516. Z stacks were taken at 1 µm steps.

### Flow cytometry

Organoids were dissociated into single cells with TrypLE Express (37 °C, 5 min) and cells were collected by centrifugation (2000rpm, 2 min). Cells were incubated in PBS with LIVE/DEAD stain (ThermoFisher) (10 min on ice), washed in FACS buffer (1% FBS in PBS), fixed in 0.5% PFA (37 °C, 15 min), permeabilised in 70% ethanol (10 min on ice), washed in FACS buffer, stained with Active Caspase-3 antibody (BD 560626) (30 min), washed and then resuspended in FACS buffer. Cells were analysed using LSRFortessa Cell Analyzer (BD Biosciences) and data analysed in FlowJo.

### Statistical analysis

All statistical analyses were performed using GraphPad Prism 6.0a (GraphPad, La Jolla, CA) for Windows. Tests performed are described in individual Figure legends, along with *p* values and significance (ns = not significant, *p < 0.05, **p < 0.01, ***p < 0.001, ****p < 0.0001).

## Electronic supplementary material

S1

S2

S3

S4

S5

Supplementary Material and Methods

Legends to Supplementary Figures
