# Supplementary figures and images for: Chir99021 and Valproic acid reduce the proliferative advantage of Apc mutant cells
Source: Cell Death Dis. 2018 Feb 15;9(3):255. doi: 10.1038/s41419-017-0199-9 (PMC5833359; doi:10.1038/s41419-017-0199-9)

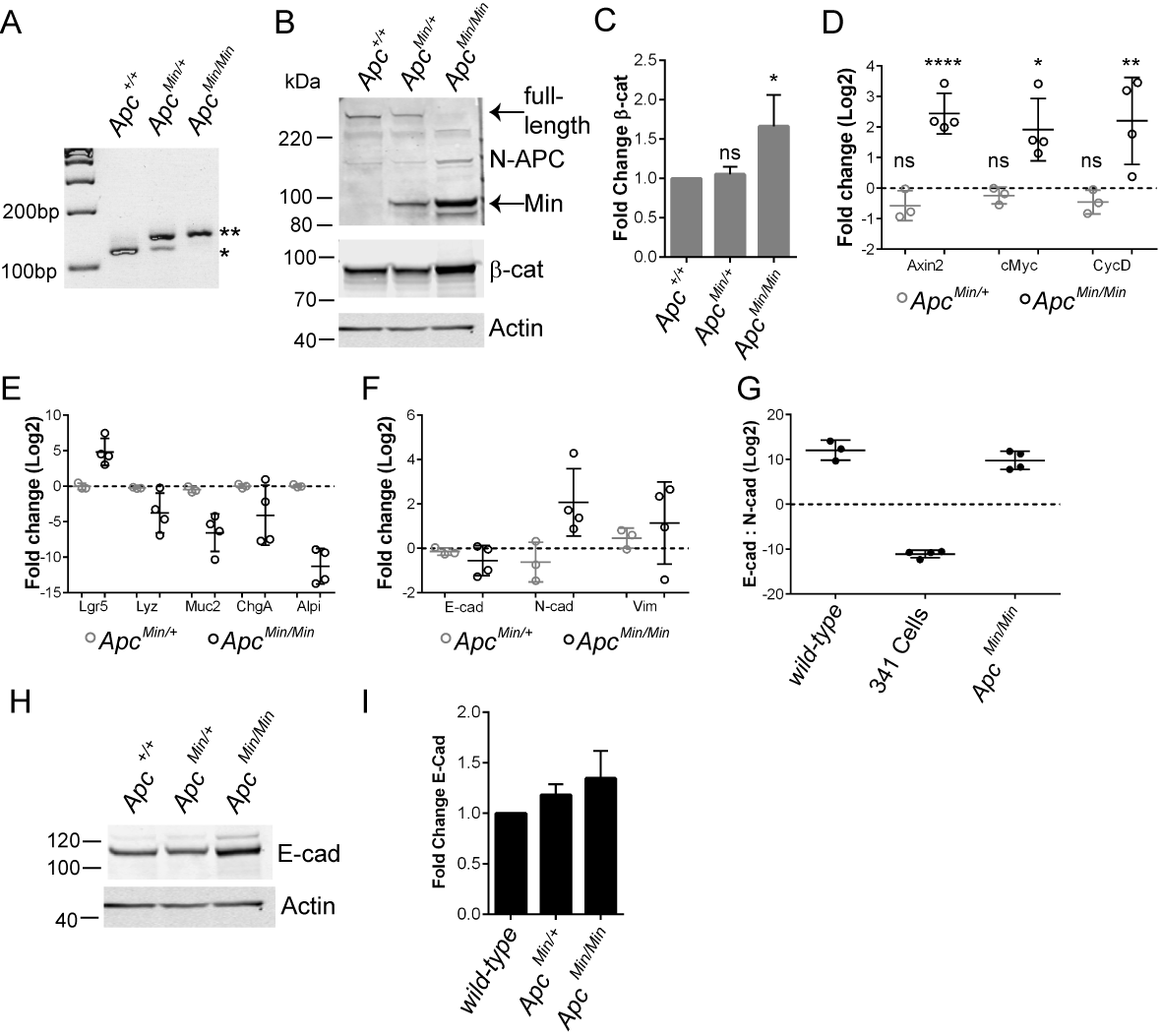

Supplement: Supplementary file 1 — S1 [file 41419_2017_199_MOESM1_ESM.tif]

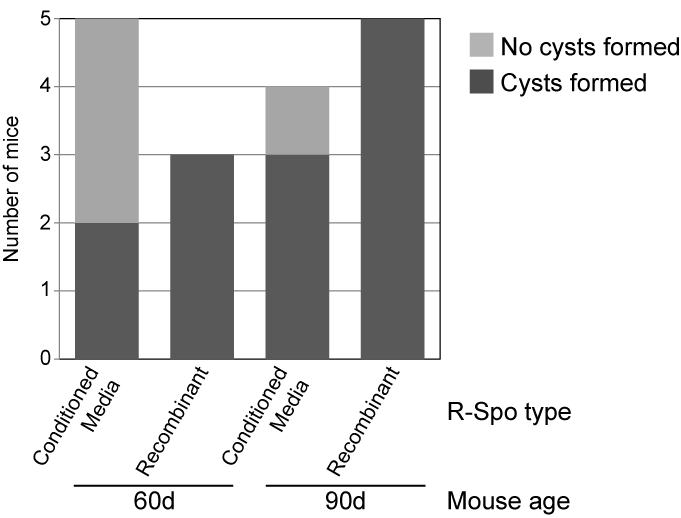

Supplement: Supplementary file 2 — S2 [file 41419_2017_199_MOESM2_ESM.tif]

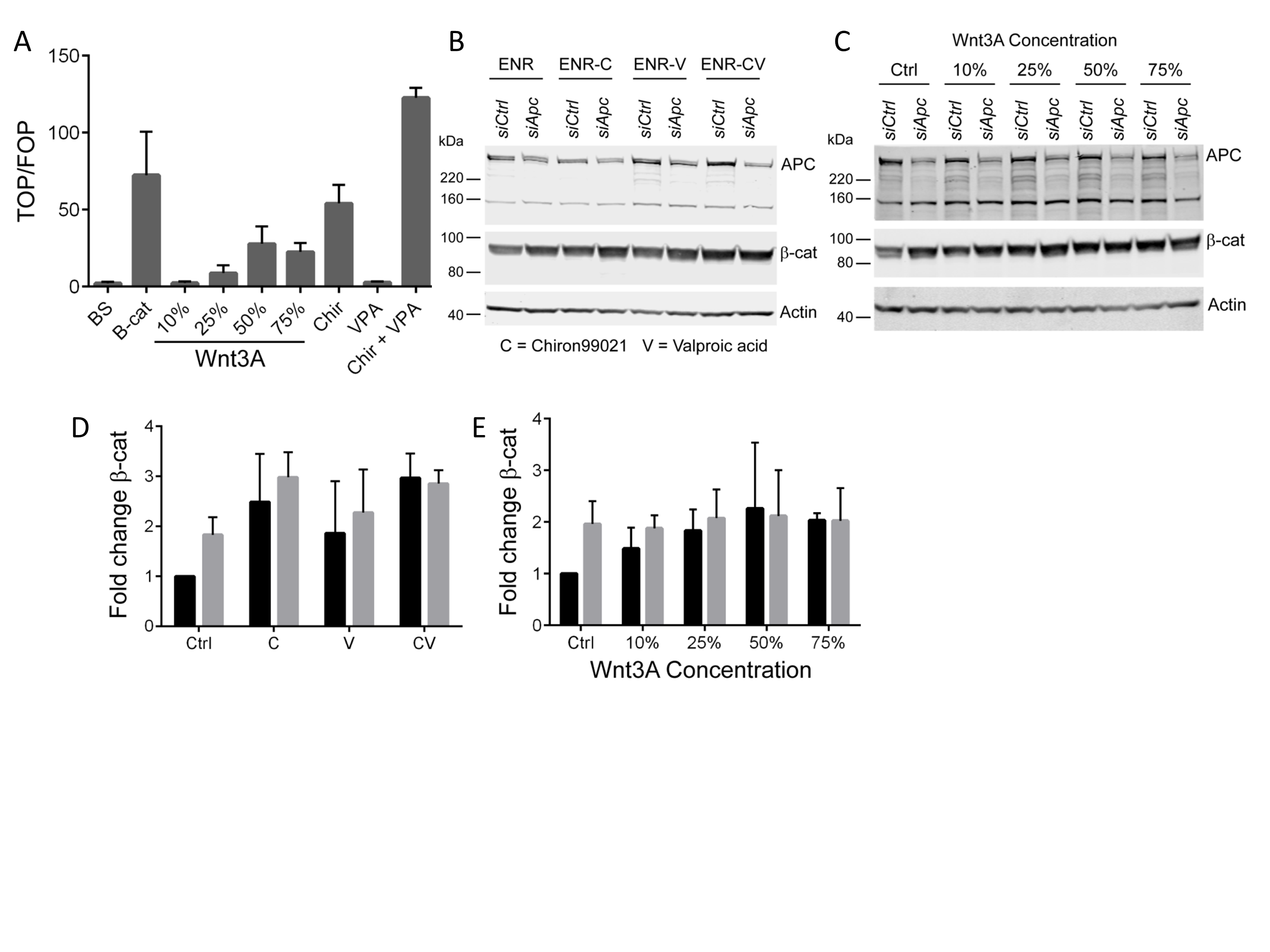

Supplement: Supplementary file 3 — S3 [file 41419_2017_199_MOESM3_ESM.tif]

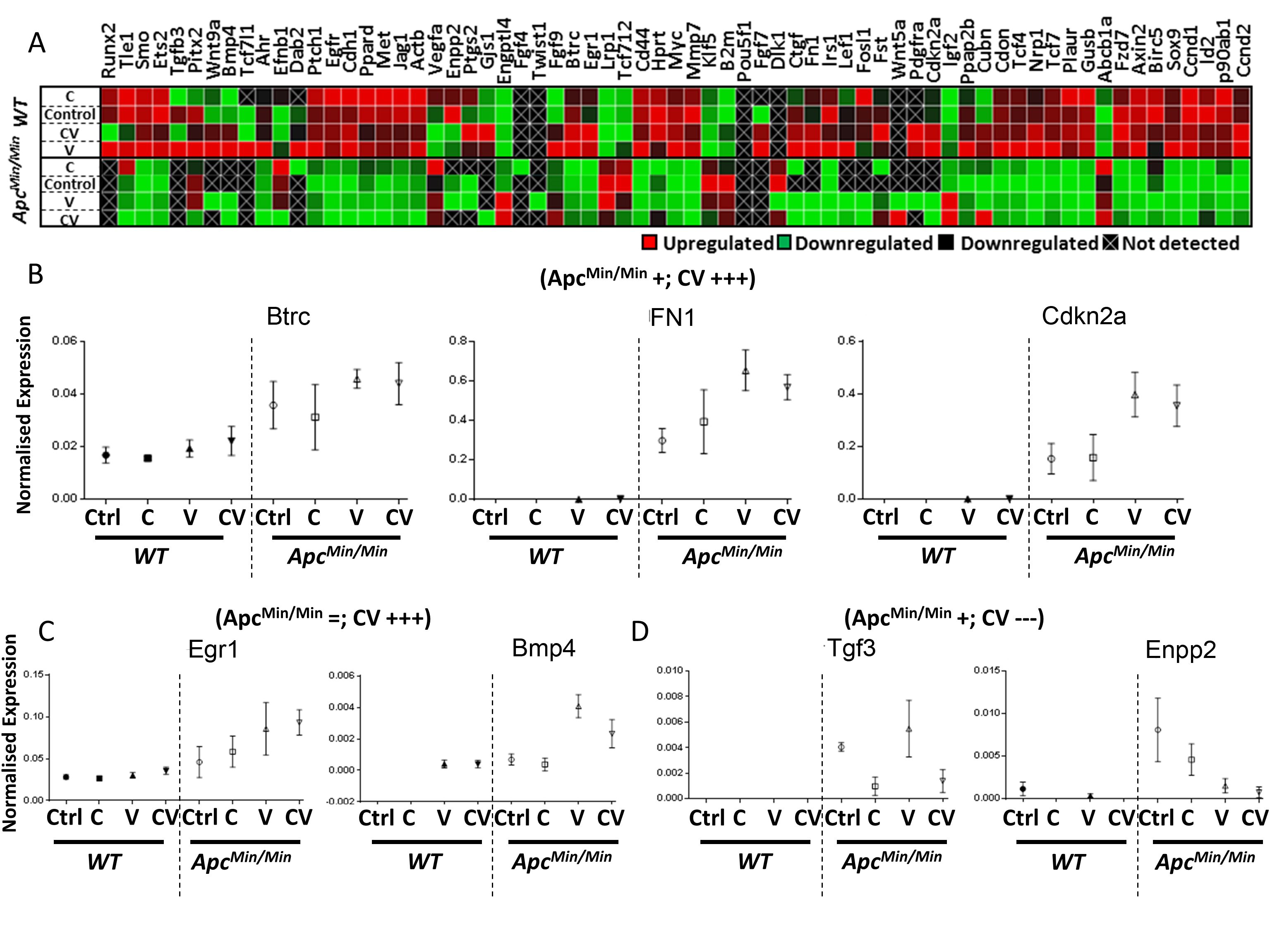

Supplement: Supplementary file 4 — S4 [file 41419_2017_199_MOESM4_ESM.tif]

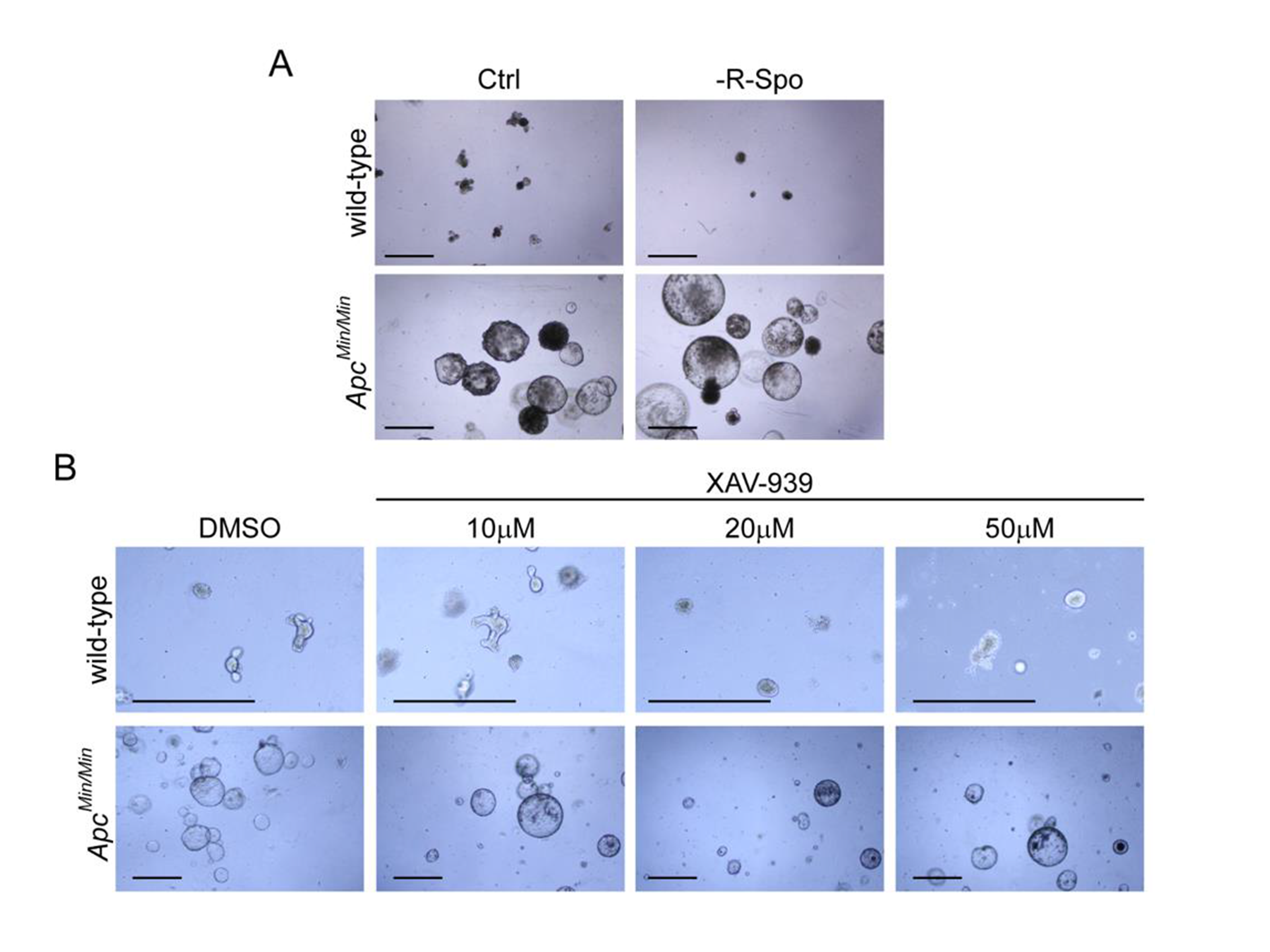

Supplement: Supplementary file 5 — S5 [file 41419_2017_199_MOESM5_ESM.tif]
