# Supplementary material for: Chir99021 and Valproic acid reduce the proliferative advantage of Apc mutant cells
Source: Cell Death Dis. 2018 Feb 15;9(3):255. doi: 10.1038/s41419-017-0199-9 (PMC5833359; doi:10.1038/s41419-017-0199-9)
Supplement: Supplementary file 6 — Supplementary Material and Methods [file 41419_2017_199_MOESM6_ESM.docx]

## Supplementary Material and Methods

### Organoid Culturing

The small intestine was removed, flushed with PBS and opened longitudinally. Tissue was washed thoroughly with PBS, incubated in 3mM EDTA in PBS (20mins) and crypts detached mechanically by vigorous shaking in PBS. Crypt suspension was washed twice in PBS then once in Advanced DMEM/F12 (ADF) before filtration through a 70µm cell strainer (Greiner, Frickenhausen, Germany). Crypts were then suspended in Growth Factor Reduced Phenol red free Matrigel (BD Biosciences, Oxford, UK). Organoids were grown in crypt media (ADF supplemented with 10mM HEPES, 2mM Glutamax, 1mM N-Acetylcysteine, N2 (Gemini, Sacramento, CA), B27 (Life Technologies), Pen/Strep (Sigma-Aldrich, St. Louis, MO)) containing growth factors (EGF (50ng/ml; Invitrogen), Noggin (100ng/ml; eBioscience) and R-Spondin, either using conditioned media (1:4) or recombinant (500ng/ml)). Additional growth factors Chiron99021 (3µm; Invitrogen, Waltham, MA), Valproic acid (1mM; Invitrogen) and Y27632 (10µm; Cambridge Bioscience, Cambridge, UK) were added for the first 48h culture. Organoids were passaged by first physically breaking up Matrigel, then washing in ADF and mechanical dissociation into individual crypts by pipetting. Individual crypts were re-suspended in Matrigel and grown in crypt media containing growth factors.

### Preparation of conditioned media

L cells for making Wnt3A Conditioned Media were obtained from ATCC, and L cells for making R-Spo Conditioned Media were a kind gift form Prof. Owen Sansom (Beatson Institute of Cancer Research, University of Glasgow). Cells were maintained in DMEM (Gibco) supplemented with 10% FBS (PAA Laboratories), 1% Pen/Strep and 0.4mg/ml G-418 (Gibco). To prepare conditioned media, cells were split 1:10 and cultured in media without G-418 for 4d (until cells were confluent). Media was removed and sterile-filtered, fresh media was added to cells and they were cultured for a further 3d. Media was removed from cells and sterile-filtered and mixed 1:1 with media from the first 4d of culture. Different batches of conditioned media were prepared so that its composition matched the different media required for cells and organoids. Specifically, conditioned media for cells, was prepared using DMEM media supplemented with 10% FBS and 1% Pen/Strep. For organoids, conditioned media was prepared using ADF base media (ADF supplemented with 1% Pen/Strep, 10mM HEPES and 2mM Glutamax), N2 and B27 were added after sterile-filtration.

### Immunofluorescence

Organoids were grown in Matrigel in 8-chamber µ slides (Ibidi, Munich, Germany) for 3-5 days and fixed in warmed 4% paraformaldehyde in PBS (pH7.4) for 20mins (37ᵒC), permeabilised for 1h in 1% Triton-X100 (this and all subsequent steps were carried out at RT), and blocked for 1h in 1% BSA, 3% normal goat serum, 0.2% Triton-X100 in PBS. Organoids were incubated in antibodies overnight in Working Buffer (0.1% BSA, 0.3% normal goat serum, 0.2% Triton-X100 in PBS), washed 5x in Working Buffer, then incubated overnight in Working Buffer containing secondary antibodies, 5µg/ml Hoechst 33342 and Phalloidin before they were washed 5x in Working Buffer and mounted in ProLong Gold antifade (Molecular Probes).

For tissue sections, small intestine and colon were isolated from mice and flushed first with PBS then with 4% paraformaldehyde (pH7.4). Tissue was opened longitudinally, washed briefly in PBS and incubated in 4% paraformaldehyde (pH7.4) at 4ᵒC overnight. A small, square piece of tissue was excised with a scalpel and embedded in 3% Low Melt Temperature Agarose. Using a Vibratome (Leica, Wetzlar, Germany) the agarose embedded tissue was sliced into 200µm thick sections. These sections were permeabilised for 2h in 2% Triton-X100 (this and all subsequent steps at 4ᵒC), blocked for 2h in 1% BSA, 3% normal goat serum, 0.2% Triton-X100 in PBS, and incubated in primary antibodies for 3 days, washed in Working Buffer (5x, 1h), incubated in secondary antibodies along with Phalloidin and Hoechst (as described above), washed in Working Buffer (5x 1h) and mounted in ProLong Gold antifade (Molecular Probes). Sections were mounted on coverslips between 120µm spacers to prevent compression and preserve tissue structure. To prepare cryo-sections, agarose-embedded tissue blocks were incubated overnight in 30% sucrose in PBS at 4ᵒC. Tissue was frozen in liquid nitrogen cooled iso-pentane and cryo-sections cut at 10µm. Sections were collected on SuperFrost-Plus slides (VWR) and air dried, rinsed with PBS (2x 10min), permeabilised in 1% Triton-X100 (20min), rinsed with PBS, blocked for 1h in 1% BSA, 3% normal goat serum, 0.2% Triton-X100, incubated in primary antibodies overnight in Working Buffer at 4ᵒC, washed in Working Buffer (5x 5min), incubated in secondary antibodies, Hoechst and Phalloidin for 3h, washed in Working Buffer (5x 5min), rinsed in PBS (3x 5min) and mounted on coverslips in ProLong Gold antifade (Molecular Probes). For both vibratome and cryo-sectioned tissue, mounting media was allowed to harden overnight before coverslips were sealed with nail polish and samples were stored at -20ᵒC.

### Antibodies

For immunofluorescence, antibodies against ZO1 (Invitrogen 40-2200), E-cadherin 24E10 (Cell Signalling Technology 3195), β4 Integrin (Abcam Ab25254), Lysozyme (Dako A0099), PH3 (Abcam Ab10543) and Ki67 (Abcam Ab16667) were used. AlexaFluor-conjugated secondary antibodies were obtained from Molecular Probes and used according to manufacturer’s instructions. DNA was visualised using 5µg/ml Hoechst 33342 (Molecular Probes), and the F-actin was detected with 0.26µM AlexaFluor-conjugated Phalloidin (Cytoskeleton). For Western blotting, antibodies against Actin C4 (Abcam Ab3280), β-cat (BD Biosciences 610154), E-cadherin 24E10 (Cell Signalling Technology 3195) and N-APC (Midgeley et al) were used. IRDye800/700-conjugated secondary antibodies were obtained from Rockland.

### qPCR primers

Primers for qPCR were obtained from Eurofins and designed utilising NCBI Primer BLAST or from previously published work as specified. Primer sequences (written 5’-3’) used for each gene are: Actin forward TTGCTGACAGGATGCAGAAG, reverse ACATCTGCTGGAAGGTGGAC; Axin2 forward AACCTATGCCCGTTTCCTCT, reverse CTGGTCACCCAACAAGGAGT; cMyc forward CACACAACGTCTTGGAACGT, reverse CGTCTGCTTGAATGGACAGG; CycD forward TCCCGCAGTGTTCCTATTTC, reverse CCAAGAAACGGTCCAGGTAA; Lgr5 forward AACGGTCCTGTGAGTCAACC, reverse CTCCTGCTCTAAGGCACCAC {Martinez Rodriguez, 2012 #2699}; Lys forward GAGACCGAAGCACCGACTATG, reverse CGGTTTTGACATTGTGTTCGC {Wang, 2011 #2700}; Muc2 forward GCTGACGAGTGGTTGGTGAATG, reverse GATGAGGTGGCAGACAGGAGAC {Wlodarska, 2011 #2701}; ChgA forward AGACTACAGACCCACTCCCG, reverse AGATGACTTCCAGGACGCAC; Alpi forward ACCCCTCAGTAGACCCTTCC, reverse CCTTGGTCAATACGACCCCC; E-cad forward ACTGTGAAGGGACGGTCAAC, reverse GGAGCAGCAGGATCAGAATC {Sheahan, 2008 #2702}; N-cad forward AGGGTGGACGTCATTGTAGC, reverse CTGTTGGGGTCTGTCAGGAT {Karabekian, 2009 #2703}; Vim forward CGGAAAGTGGAATCCTTGCA, reverse CACATCGATCTGGACATGCTGT {Yates, 2007 #2704}.
