# Supplementary material for: Chir99021 and Valproic acid reduce the proliferative advantage of Apc mutant cells
Source: Cell Death Dis. 2018 Feb 15;9(3):255. doi: 10.1038/s41419-017-0199-9 (PMC5833359; doi:10.1038/s41419-017-0199-9)
Supplement: Supplementary file 7 — Legends to Supplementary Figures [file 41419_2017_199_MOESM7_ESM.docx]

**Legends to Supplementary Figures**

**Figure S1. Expression of Wnt targets, differentiation markers and EMT markers in *wild-type*, *Apc^Min/+^*, and *Apc^Min/Min^* organoids**

(A) Genotyping confirms that transformed organoids have undergone LOH: only the 123bp band (**) corresponding to *Apc^Min^* is present not the smaller band reflecting the wild-type allele (*).

(B) Western blot comparing wild-type, *Apc^Min/+^* and *Apc^Min/Min^* organoids shows only truncated (~90kDa) APC is present in *Apc^Min/Min^* cysts, in contrast to wild-type and *Apc^Min/+^* organoids where the ~300kDa full-length form of APC is detectable.

(C) β-catenin protein is more abundant in *Apc^Min/Min^* cysts than in wild-type, as confirmed by quantitation (average value from 3 independent experiments, ± SD).

(D-F) qPCR analysis showing Log2 fold change in *Apc^Min/+^* and *Apc^Min/Min^* organoids compared to wild-type. (D) Expression of Wnt target genes was increased in *Apc^Min/Min^* but not *Apc^Min/+^*.

(E) Expression of differentiated cell markers *Lyz*, *Muc2*, *ChgA* and *Alpi* associated with Paneth cells, Goblet cells, enteroendocrine cells and enterocytes, respectively, were all reduced in *Apc^Min/Min^* cysts compared to wild-type. Expression of *Lgr5* (marking undifferentiated cells including stem cells) was increased in *Apc^Min/Min^* cysts. Expression of all markers was unchanged in *Apc^Min/+^* organoids compared to wild-type.

(F) Increased expression of the mesenchymal markers *Ncad* and *Vim*  was observed in *Apc^Min/Min^* cysts only.

(G) The ratio of *Ecad:Ncad* mRNA was similar in *Apc^Min/Min^* cysts to that in wild-type organoids, whilst truly mesenchymal 341 cells (mouse fibroblasts) show a much lower *Ecad:Ncad* ratio.

(H) Immunoblotting lysates from wild-type, *Apc^Min/+^* and *Apc^Min/Min^* organoids and quantitating (I) relative abundance of E-cadherin revealed that E-cadherin expression is slightly but not significantly increased in *Apc^Min/Min^* cysts.

**Figure S2. Organoids derived from older mice are more likely to grow with cyst morphology**

Organoid cultures were prepared from individual *Apc^Min/+^* mice aged 60 or 90 days and grown in ENR media containing either R-spondin conditioned media or recombinant R-spondin. The number of cultures that developed organoids with cyst morphology was scored. Two of five organoid cultures derived from 60d old *Apc^Min/+^* mice (and grown in the presence of R-Spo-conditioned media) developed organoids with cyst morphology. Three out of four organoid cultures derived from 90d old *Apc^Min/+^* mice (and grown in R-Spo conditioned media) developed organoids with cyst morphology. All organoid cultures derived from *Apc^Min/+^* mice and grown in recombinant R-Spo developed organoids with cyst morphology.

**Figure S3. Wnt responses are increased by Chir and Valproic acid and by exogenous Wnt3A**

(A) TOP/FOP assay of HEK293 cells reveals increased Wnt signalling responses in Wnt3A conditioned media, and Chiron99021 alone or in combination with Valproic acid. However, Valproic acid alone did not increase the Wnt response. BS is an empty vector acting as negative control, β-cat was introduced along with TOP-Renilla as a positive control (B-cat). Averages of three independent transfections are shown ±SD.

(B-E) Western blot analysis (B, C) and quantification (D, E) of β-catenin protein in HAB92 cells transfected with *Apc* or *Ctrl* siRNAs and grown in the presence of Chiron99021 and/or Valproic acid (B, D); or in different concentrations of Wnt3A-conditioned media (C, E). β-catenin protein level was increased in APC depleted cells by Chiron99021 alone (p=0.037), or in combination with Valproic acid (p=0.019). Only combined Chiron99021 and Valproic acid treatment significantly increased β-catenin protein in *ctrl* siRNA transfected cells (p=0.020). Wnt3A-conditioned media did not significantly increase β-catenin protein in *Apc* siRNA transfected cells (p>0.05 for all concentrations), but 75% Wnt3A conditioned media did significantly increase β-catenin in C*trl* siRNA transfected cells (p=0.006). Average of three independent knock-down experiments are shown ±SD (D, E).

**Figure S4. Differential transcription of Wnt target genes**

(A) The transcription of Wnt target genes was measured in wild-type and *Apc^Min/Min^* organoids grown in control media (ENR) or in media containing Chiron99021 and Valproic acid (ENR-CV). Values of three biological samples are plotted as a heat map with increasing values reflected by colour changes from green (lower) to black (unchanged), to red (higher), relative to housekeeping gene controls (GAPDH, Tbp) Black with a white cross indicates samples that were below the detection limit.

(B) qPCR data for individual Wnt targets shown in panel A that were upregulated in *Apc^Min/Min^* compared to wild-type organoids and further upregulated in ENR-CV. Data is shown normalized to actin in each panel. Shown are the averages of 3 biological replicates ±SD. Upregulation of these genes is normally associated with increased proliferation and does not explain the reduced growth of *Apc^Min/Min^* cysts. However, it is possible that over activation of these pathways causes senescence.

(C) qPCR data for individual Wnt targets shown in panel A that were upregulated in ENR-CV but not in untreated *Apc^Min/Min^* cysts. Data is shown normalised to actin in each panel. Shown are the averages of 3 biological replicates ±SD. BMP4 can contribute to decreased proliferation of cells and is associated with differentiation in the intestinal crypt axis [36]. However, in other situations BMP4 can also stimulate cell proliferation suggesting that its effects are context-dependent [37]. Egr1 is associated with increased proliferation and/or migration making it unlikely that it contributes to the observed phenotype [38].

(D) qPCR data for the individual Wnt targets shown in panel A that were upregulated in *Apc^Min/Min^* cysts compared to wild-type but were then significantly downregulated after ENR-CV treatment. Data is shown normalized to actin in each panel. Shown are the averages of 3 biological replicates ±SD. Both of these genes are related to inflammatory changes: a reduction in *Tgfb3* has been detected in experimentally induced intestinal inflammation [39]. This decrease is predicted to promote cell cycle progression; however, the effects of TGF are pleitropic [40]. Enpp2 is involved in the production of lysophosphatidic acid, a potent mitogen and stimulator of migration [41].

**Figure S5. *Apc^Min/Min^* organoids are unaffected by Wnt depletion**

(A) Wild-type organoids g

row in the presence of R-Spo (top left panel), but not in its absence (top right). In contrast, *Apc^Min/Min^* cysts grow similarly in the presence (bottom left) or absence (bottom right) of R-Spo.

(B) Wild-type organoids (top row) grown in the presence of XAV-939 at concentrations of 20µM and higher did not undergo branching, remaining small and circular. *Apc^Min/Min^* cysts (bottom row) are unaffected by increasing concentrations of XAV-939. Scale bars = 500µm.

**References**

36. Ampuja M, Jokimaki R, Juuti-Uusitalo K, Rodriguez-Martinez A, Alarmo EL, Kallioniemi A. BMP4 inhibits the proliferation of breast cancer cells and induces an MMP-dependent migratory phenotype in MDA-MB-231 cells in 3D environment. *BMC Cancer* 2013; **13**: 429.

37. Lorente-Trigos A, Varnat F, Melotti A, Ruiz i Altaba A. BMP signaling promotes the growth of primary human colon carcinomas in vivo. *J Mol Cell Biol* 2010; **2**: 318-332.

38. Ben-Chetrit N, Tarcic G, Yarden Y. ERK-ERF-EGR1, a novel switch underlying acquisition of a motile phenotype. *Cell adhesion & migration* 2013; **7**: 33-37.

39. Suzuki R, Miyamoto S, Yasui Y, Sugie S, Tanaka T. Global gene expression analysis of the mouse colonic mucosa treated with azoxymethane and dextran sodium sulfate. *BMC Cancer* 2007; **7**: 84.

40. Potten CS, Booth C, Pritchard DM. The intestinal epithelial stem cell: the mucosal governor. *Int J Exp Pathol* 1997; **78**: 219-243.

41. Jethwa SA, Leah EJ, Zhang Q, Bright NA, Oxley D, Bootman MD *et al*. Exosomes bind to autotaxin and act as a physiological delivery mechanism to stimulate LPA receptor signalling in cells. *Journal of cell science* 2016; **129**: 3948-3957.
